# Epidemiological and demographic drivers of ischemic stroke attributed to high fasting plasma glucose from 1990 to 2021: findings from the 2021 global burden of disease study

**DOI:** 10.3389/fpubh.2025.1511518

**Published:** 2025-05-19

**Authors:** Yanwen Dong, Yangyang Wang, Xiaomei Lan, Huiyan Zeng

**Affiliations:** ^1^Guangzhou University of Chinese Medicine, Guangzhou, China; ^2^The Second Clinical College of Guangzhou University of Chinese Medicine, Guangzhou, China; ^3^The Affiliated Huizhou Hospital, Guangzhou Medical University, Huizhou, China; ^4^Guangzhou Development District Hospital, Guangzhou, China; ^5^The Second Affiliated Hospital of Guangzhou University of Chinese Medicine, Guangzhou, China

**Keywords:** hyperglycemia, ischemic stroke, health inequity, disease burden, visualization platform, GBD

## Abstract

**Background:**

This study aims to analyze the global, regional, and national burden of Ischemic Stroke (IS) attributed to High Fasting Plasma Glucose (HFPG) from 1990 to 2021, identify risk sources in different areas, and develop a platform to assess the disease burden in 204 countries and regions.

**Methods:**

Using data from the 2021 Global Burden of Disease study, we analyzed IS-related deaths and disability-adjusted life years (DALYs) attributed to hyperglycemia from 1990 to 2021. We conducted detailed analyses by region, gender, and age. In different Socio-demographic Index (SDI) regions, we used the Age-Period-Cohort (APC) model to evaluate the impact of age, cohort, and period on Age-Standardized Mortality Rate (ASMR) and decomposition analysis to separate the contributions of population, aging, and epidemiological changes. A visualization platform was built using the Shiny package.

**Results:**

In 2021, approximately 18% of all IS-related DALYs and deaths were attributed to HFPG, with an annual percentage change (EAPC) in DALYs of −0.715 (−0.811, −0.620) and deaths of −0.959 (−1.059, −0.859). The primary mortality group was aged 80–84. ASMR, categorized by SDI, showed increases in Low-middle SDI: 0.482 (0.422, 0.542) and Low SDI: 0.287 (0.218, 0.356), particularly in Central Asia, East Asia, North Africa and Middle East, and South Asia. The APC model indicates that age is the primary source of burden in High, High-middle, and Middle SDI regions, with ASMR trends improving over the last 5 years, contrary to trends in Low-middle and Low SDI regions. Decomposition analysis suggests that aging and epidemiological changes in High-middle and High SDI regions outweigh population growth. In contrast, in low, low-middle, and middle SDI regions, the population remains the most significant influence, with the impact of aging increasing. The HFPG-IS platform is accessible at http://116.196.73.86:3838/GBD/HFPG-IS/.

**Conclusion:**

There is a significant imbalance in IS health attributed to HFPG globally. In low SDI regions, larger populations face more uneven healthcare distribution, necessitating improvements in healthcare infrastructure, especially in areas like the United Arab Emirates. There should be a focus on metabolic adjustments and attention to high-risk groups, such as those aged 80–84, to reduce health losses.

## 1 Introduction

Over the past 50 years, rapid advancements in healthcare and significant lifestyle changes have fundamentally shifted health priorities in most regions worldwide (1). According to the 2017 Global Burden of Disease (GBD) study, stroke ranked as the third leading cause of death and disability globally and the second leading cause of death (2). The increasing prevalence of diabetes (3) has led to a growing burden of diseases attributed to high blood sugar (4). Research indicates that from 1990 to 2019, the contribution of High Fasting Plasma Glucose (HFPG) to DALYs attributed to stroke increased by 40.3% (5). The WHO suggests that effective stroke prevention strategies should focus on reducing risks associated with diabetes (high fasting blood glucose), unhealthy diets, and high BMI (6).

Research has shown that the global increase in the burden of ischemic stroke (IS) patients is linked to elevated fasting blood glucose. Patients with risk factors like high blood sugar have a significantly increased susceptibility to IS. HFPG contributes to systemic vascular dysfunction, which may lead to complications such as ischemic stroke and myocardial infarction. Interventions with metformin or other antidiabetic medications can effectively reduce the incidence of IS (7). However, few studies have explored the burden and trends of IS caused by metabolic factors, particularly those attributed to high blood glucose.

This study utilizes data from the 2021 Global Burden of Disease (GBD 2021) to analyze the distribution patterns and trends of IS attributed to high blood sugar across different times, locations, and demographic groups. It also identifies regional risk factors. These insights can enhance the allocation of healthcare resources and inform policy-making.

## 2 Materials and methods

### 2.1 Data sources

This study analyzes data from the 2021 GBD study, accessible via the Global Health Data Exchange (GHDx) website.^1^ Since 1990, GBD has employed standardized methodologies to estimate mortality and health loss for various diseases, updating results every 2–3 years as part of ongoing quality improvement (8, 9). GBD 2021 offers the latest dataset on the global burden of IS from 1990 to 2021 across 204 countries and regions. This cycle incorporates new data sources and improved methods to provide updated estimates compared to previous GBD studies (10). The GBD database includes diverse raw data from surveys, censuses, and other health-related sources. Mortality data is estimated using the Cause of Death Ensemble Model (CODEm), which integrates statistical models to validate cause-specific mortality (11). The calculation of mortality and disability-adjusted life years (DALYs) involves summing the years of life lost (YLL) due to premature death and years lived with disability (YLD) for each age, gender, and location (12). Mortality and DALY rates are derived from the average of 1,000 draws, with 95% uncertainty intervals (UI) calculated to estimate disease burden. UIs account for variance in parameter estimates data, collection, and model selection uncertainties.

### 2.2 Risk factors and definition of elevated fasting blood glucose

The GBD study categorizes risk factors into environmental, behavioral, and metabolic. Metabolic risk factors related to IS include high fasting plasma glucose (HFPG). The GBD uses HFPG, defined as any level above the theoretical minimum risk exposure level [4.8–5.4 mmol/L], as an individual risk factor to estimate disease burden (13). According to the GBD framework, the disease burden from HFPG is observed only in individuals over 25.

### 2.3 Data analysis

For data analysis, the 2021 GBD study divided the world into 21 regions based on epidemiological similarities and geographical proximity. Additionally, the study provided the SDI, a comprehensive indicator of the socio-economic conditions affecting health outcomes. Countries and regions were classified into five SDI quintiles: high, high-middle, middle, low-middle, and low development levels.

From the GBD 2021 study, key metrics such as the age-standardized rate (ASR), mortality figures, percentages, and DALYs were extracted. ASR is a metric adjusted for differences in age distribution across populations or time periods, ensuring comparability. It is calculated as follows:


$$ASR=\underset{i}{\sum }w_{i}\times r_{i}$$

where *w_i_* represents the proportion of the standard population in age group *i*, and *r_i_* denotes the corresponding rate.

To quantify trends in IS burden attributable to HFPG, the estimated annual percentage change (EAPC) was computed for both the age-standardized mortality rate (ASMR) and age-standardized DALYs rate (ASDR).

The EAPC was estimated using a generalized linear model with a Gaussian distribution, defined as:


$$\mathrm{EAPC}=\left(e^{\beta }-1\right)\times 100$$

where *β* is the slope coefficient derived from the linear regression model, with time (years) as the independent variable:

ln(R)=β×year+α

where *R* represents ASMR or ASDR. A higher EAPC indicates a more significant disease burden of IS attributed to HFPG. If both the EAPC and the lower limit of its 95% confidence interval (CI) are more significant than 0, the disease burden is increasing; otherwise, it suggests a declining trend.

Through regional decomposition analysis, we utilized hierarchical clustering to categorize the demographic and epidemiological factors that had an impact on the mortality of IS cases attributed to HFPG spanning from 1990 to 2021. This allowed us to assess the contributions of population growth, aging, and epidemiological changes to the ASMR across different SDI regions. Subsequently, we used Spearman correlation analysis to examine the relationship between ASMR and SDI.

Frontier analysis identifies leading countries or regions at the forefront and driving progress. These areas exhibit the lowest burden of IS attributed to HFPG relative to their SDI level. The “efficiency gap” refers to the difference between the observed burden in a country or region and the potentially achievable burden at their SDI. This gap can be reduced or eliminated based on the sociodemographic resources available (14).

Data analysis and visualization were conducted using R software version 4.0.2. The R packages utilized in our study included “factoextra” (15), “tidyverse” (16), “ggplot2” (17), and “stats” (18). A *p*-value of less than 0.05 was considered statistically significant.

### 2.4 HFPG-IS platform development

Given the varying disease burdens and trends across countries and regions, this study developed the HFPG-IS platform using the Shiny package (19). This platform visualizes disease burden and future trends for 204 countries and regions, allowing users to select locations and evaluation metrics.

## 3 Results

### 3.1 Global estimates of DALYs and mortality induced by HFPG-related IS

In 2021, approximately 18% of all IS-related DALYs were attributed to HFPG. DALY cases nearly doubled, increasing by 99.87% from 6.18 million in 1990 to 12.37 million in 2021. However, despite the rise in absolute numbers, the incidence rate per 100,000 decreased from 176.87 (138.36, 222.99) to 147.07 (113.94, 183.06). This suggests that a growing population may contribute to the decline in relative rates, even as absolute numbers rise. The estimated annual percentage change (EAPC) was −0.715% (−0.811, −0.62%), indicating a decline in the burden of DALYs of IS attributed to HFPG globally. The relevant data can be found in Supplementary Table1. Furthermore, approximately 18% of deaths from IS worldwide are attributed with HFPG. Over the past 30 years, the number of deaths due to IS attributed to HFPG has increased by 98.02%. However, from 1990 to 2021, the EAPC annual percentage change in deaths due to IS attributed with HFPG is −0.959% (−1.059, −0.859%), which also indicates a downward trend in the burden of deaths from IS attributed to HFPG worldwide. For specific data, please refer to Supplementary Table2.

### 3.2 Regional burden of HFPG-induced IS

Interestingly, while the global burden of IS attributed to HFPG in terms of DALYs and deaths has eased, regional differences persist. In regions with low-middle and low SDI, the EAPC indicates an ongoing increase in DALYs [Low-middle SDI: 0.51 (0.461, 0.56), Low SDI: 0.241 (0.185, 0.297)] and deaths [Low-middle SDI: 0.482 (0.422, 0.542), Low SDI: 0.287 (0.218, 0.356)], particularly in Central Asia, East Asia, North Africa and the Middle East, and South Asia. For the detailed information, please refer to Supplementary Files.

**Figure 1:**
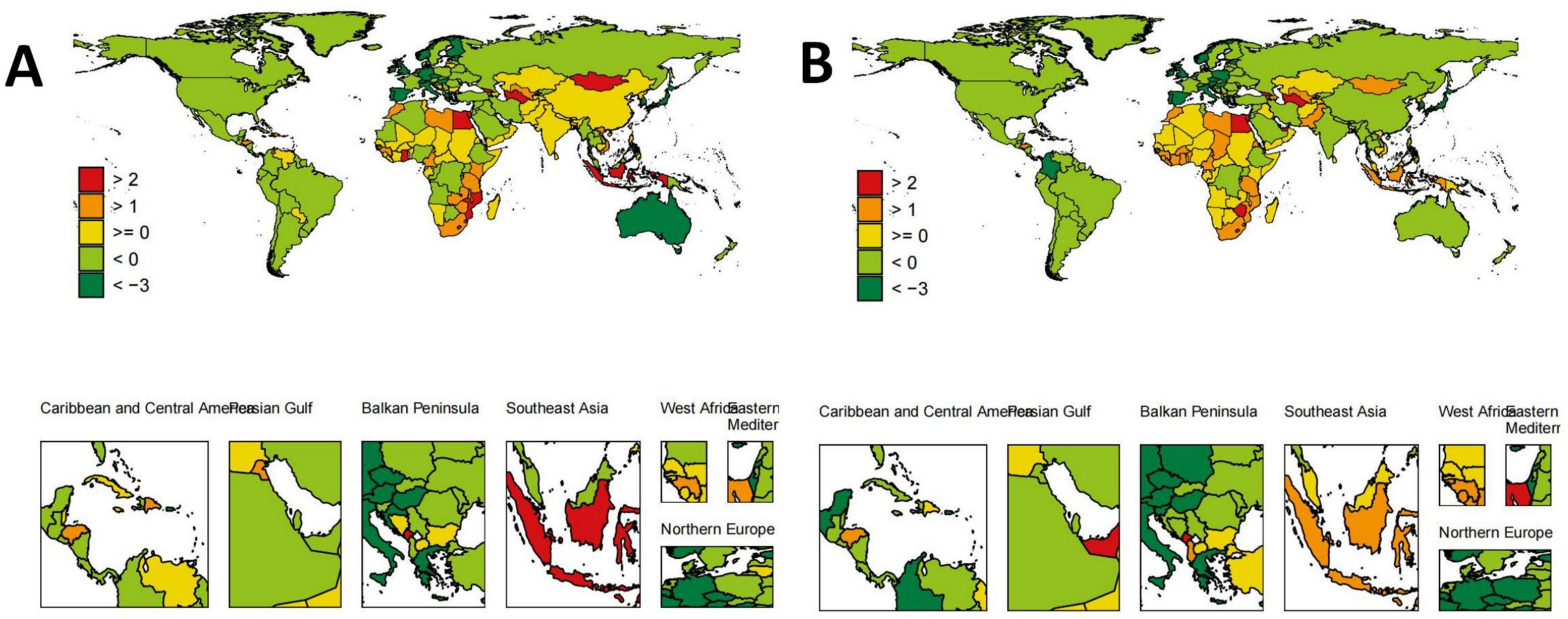
Global distribution of ASDR trends (EAPC) for HFPG-IS population by sex. **(A)** Global distribution of ASDR trends (EAPC) for male HFPG-IS population. **(B)** Global distribution of ASDR trends (EAPC) for female HFPG-IS population.

**Figure 2:**
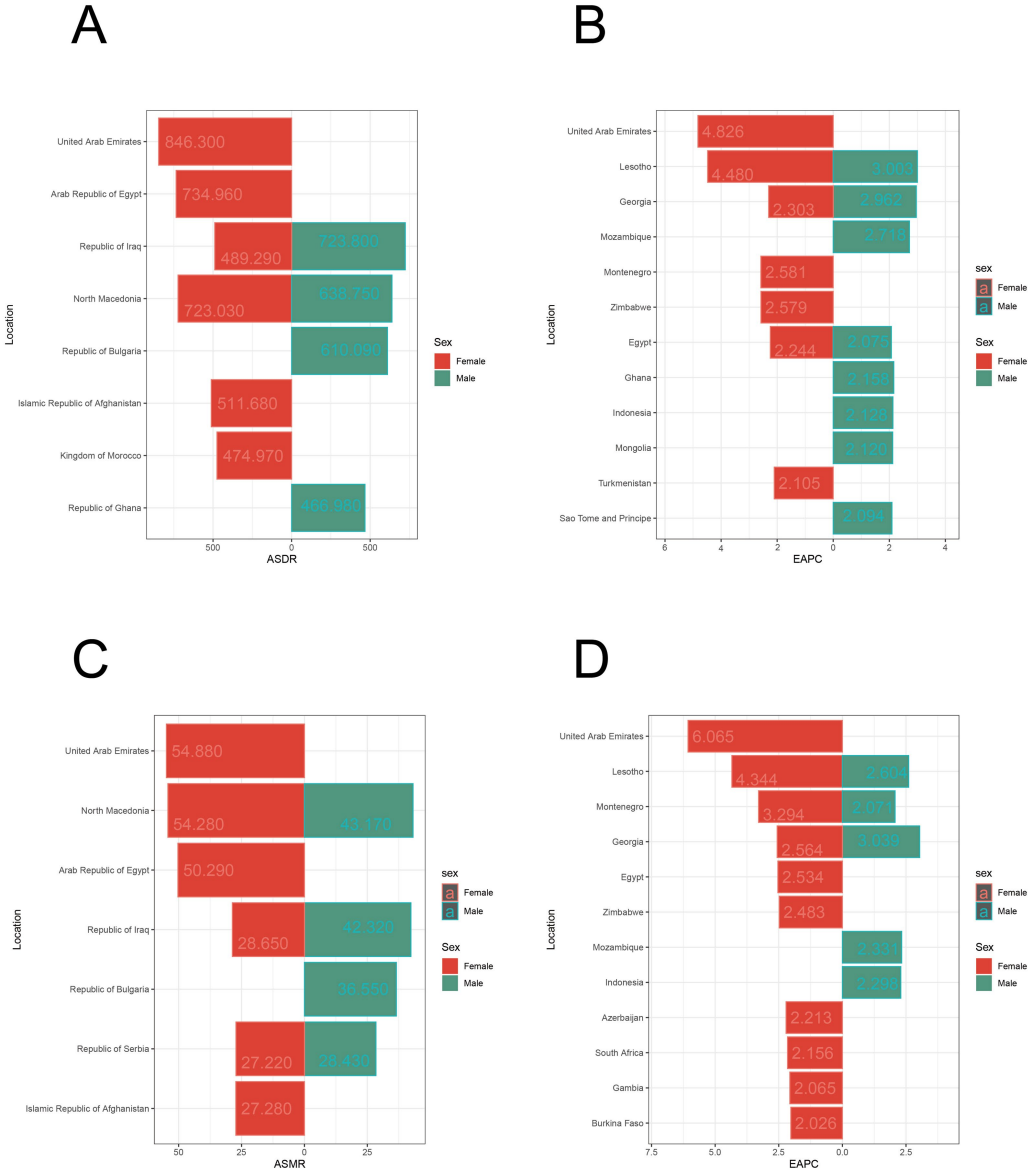
Distribution and trends of ASDR and ASMR for regional populations by sex. **(A)** Top ten regions by ASDR for population. **(B)** Top ten regions with the fastest ASDR trend (EAPC) for population. **(C)** Top ten regions by ASMR for population. **(D)** Top ten regions with the fastest ASMR trend (EAPC) for population.

As shown in Figure 1, the ASDR and EAPC for IS attributed to HFPG varied globally in 2021. Among females, the highest ASDR was observed in the United Arab Emirates [846.30 per 100,000 (612.12, 1105.56)], followed by the Arab Republic of Egypt [734.96 per 100,000 (531.74, 963.36)], males in Iraq [723.88 per 100,000 (496.62, 967.71)], and females in North Macedonia [723.03 per 100,000 (530.19, 925.63)] (Figure 2A). The fastest growth in the burden of IS attributed to HFPG was among females in the United Arab Emirates [ASDR EAPC: 4.83 (4.06, 5.59)], females in Lesotho [ASDR EAPC: 4.48 (3.84, 5.12)] and males [ASDR EAPC: 3.00 (2.64, 3.36)], and males in Georgia [ASDR EAPC: 2.96 (2.51, 3.41)] (Figure 2B).

Similarly, the ASMR and corresponding EAPC for IS attributed to HFPG varied globally in 2021. The highest ASMR was observed among females in the United Arab Emirates [54.88 per 100,000 (39.74, 71.59)], followed by females in North Macedonia [34.82 per 100,000 (26.03, 44.83)], females in the Arab Republic of Egypt [50.29 per 100,000 (36.95, 65.4)], and males in North Macedonia [43.17 per 100,000 (30.84, 55.14)] (Figure 2C). The fastest growth in the burden of IS attributed to HFPG was among females in the United Arab Emirates [ASMR EAPC: 6.07 (5.14, 7)], females in Lesotho [ASMR EAPC: 4.34 (3.67, 5.02)], and females in Montenegro [ASMR EAPC: 3.29 (3.01, 3.58)] (Figure 2D).

### 3.3 Disease burden across age groups

**Figure 3:**
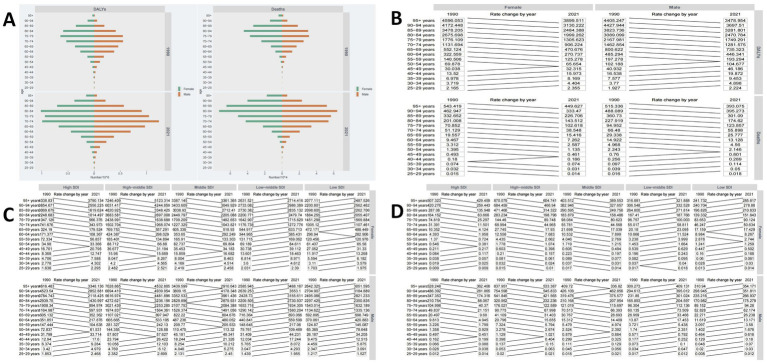
Disease burden across different age groups. **(A)** Global burden of DALYs and deaths across different age groups. **(B)** Trends in ASDR and ASMR across different age groups from 1990 to 2021. **(C)** Trends in ASDR across different age groups in low-to-high SDI regions from 1990 to 2021. **(D)** Trends in ASMR across different age groups in low-to-high SDI regions from 1990 to 2021.

In this study, the age group with the highest DALYs due to IS attributed to HFPG in 1990 was 75–79 years, while the highest mortality was in the 80–84 age group, predominantly among females. By 2021, the 80–84 age group remained the primary source of DALYs and deaths, with a trend toward a higher proportion of males (Figure 3A).

Over the past 30 years, globally, both male and female ASDR and ASMR from IS attributed to HFPG have generally decreased in older age groups (55 and above) and increased in younger age groups (below 40–44) (Figure 3B). Significant differences exist across SDI regions. In High, High-middle, and Middle SDI areas, ASMR for IS attributed to HFPG generally declined in those over 50 but increased in the 25–49 age group. In contrast, in Low-middle and low-SDI regions, the ASDR burden rose across nearly all age groups (Figure 3C). This trend is more pronounced in mortality burdens, with High, High-middle, and Middle SDI regions achieving comprehensive control over the past 30 years, while Low-middle and Low SDI regions continue to see growth (Figure 3D).

### 3.4 Impact of age, period, and cohort on ASMR

The APC model estimates age, period, and cohort effects using SDI quintiles. The age effect is represented by a longitudinal age curve, illustrating the natural history of ASR. The period effect indicates the relative risk of occurrence over time, allowing for trend tracking. Additionally, the cohort effect shows the relative incidence risk in birth cohorts, enabling observation of incidence changes. Overall, age, period, and cohort effects display consistent trends globally and across different SDI regions.

**Figure 4:**
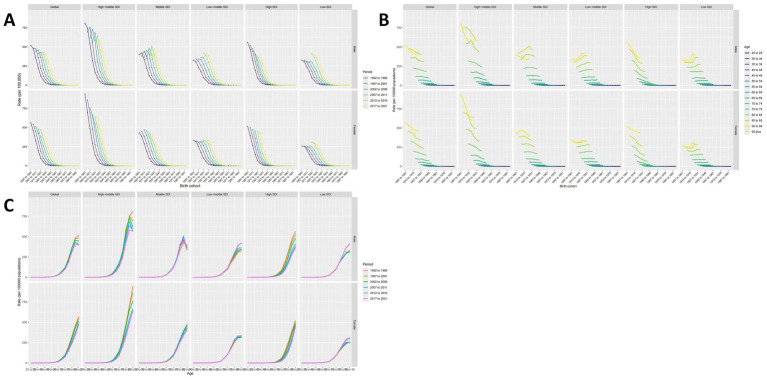
The impact of age, period, and cohort (APC) on ASMR. **(A)** Period-cohort effects. **(B)** Age-period effects. **(C)** Age-cohort effects.

The period-cohort model indicates that, throughout the study period, birth cohort effects are globally similar, generally showing a declining trend (Figure 4A), with minor fluctuations in the High-middle and Middle SDI regions. Moreover, the period effect trend is more pronounced, with ASMR gradually increasing over the past 30 years without significant shifts (Figure 4A).

The age-cohort model (Figure 4B) demonstrates that ASMR increases with age, a finding corroborated by the age-period model (Figure 4C), indicating that advanced age is a critical factor in the mortality burden of IS attributed to HFPG. Notably, in High-middle and Middle SDI regions, patients over 95 have a lower ASMR, a consistent trend across various cohorts and periods. Furthermore, ASMR showed the most favorable trends in the High, High-middle, and Middle SDI regions over the past 5 years. In contrast, the opposite was true for Low-middle and Low SDI regions (Figure 4C), highlighting a significant imbalance in the application of medical technologies, with less developed areas not benefiting adequately.

### 3.5 Analysis of the correlation between SDI and ASMR

**Figure 5:**
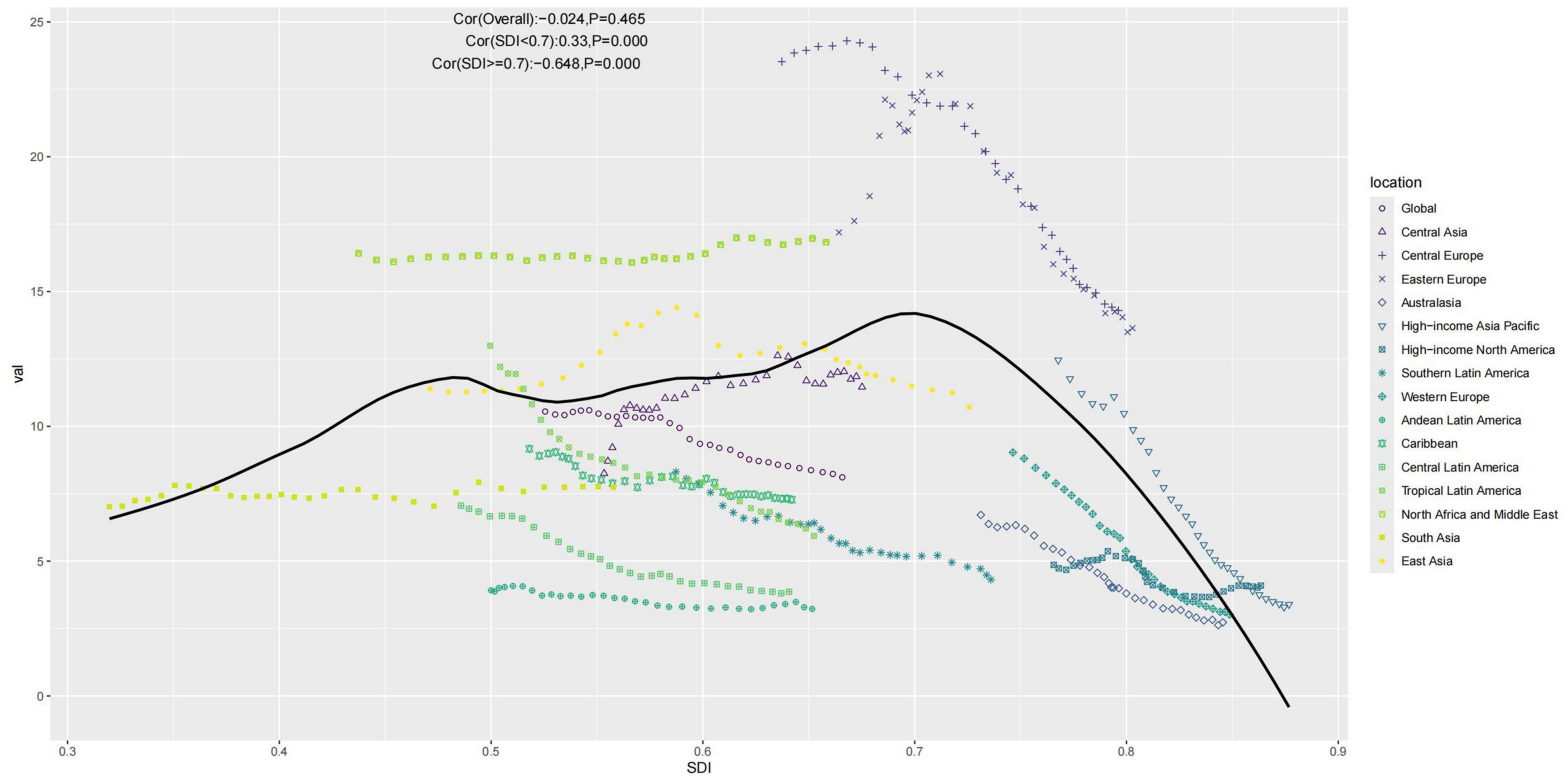
Scatter plot of ASMR in relation to SDI (Note: Points represent the mapping of SDI and ASMR for each location from 1990 to 2021, and the curve represents the fitted line).

A Spearman correlation analysis was conducted to examine the correlation between ASMR and SDI further. At a global level, there was no significant correlation between ASMR and SDI (cor = −0.024, *p* = 0.465 > 0.05). However, as shown in Figures 5, a nonlinear relationship exists between ASMR and SDI. Using an SDI value of 0.7 as a threshold, when SDI is less than 0.7, there is a positive correlation with ASMR (cor = 0.33, *p* = 0.000 < 0.001). Conversely, when SDI is 0.7 or greater, a negative correlation with ASMR is observed (cor = −0.648, *p* = 0.000 < 0.001).

### 3.6 Frontier analysis

**Figure 6:**
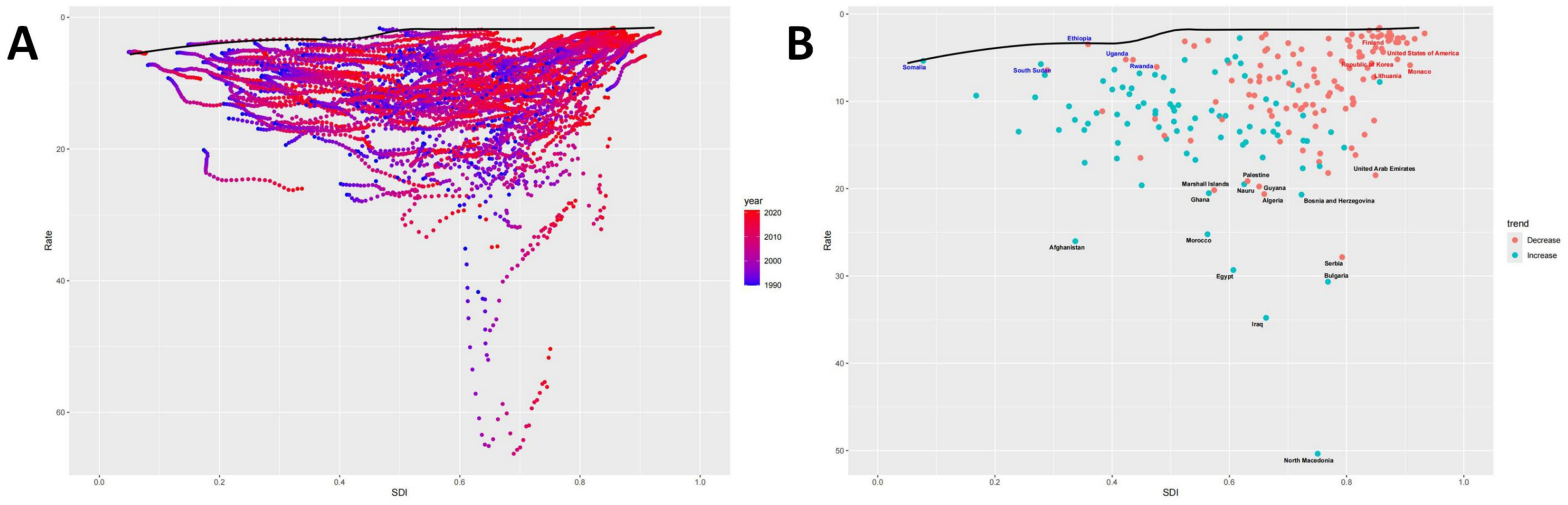
Frontier analysis. **(A)** The relationship between SDI and ASMR for each region from 1990 to 2021. Points indicate the correspondence between SDI and ASMR for each region during this period, while the curve represents the ideal ASMR at a specific SDI. **(B)** Trends in ASMR compared to 1990 for each region in 2021. Points show the ASMR trends for each region in 2021 compared to 1990, with blue indicating an increase and red indicating a decrease, and the curve representing the ideal ASMR at a specified SDI.

As the SDI increases, the effective differences for a given SDI tend to decrease with less variance, indicating stable ASMR control in high SDI regions. Conversely, regions with low SDI may benefit from enhanced control measures (Figure 6A). The top 10 countries with the most effective differences compared to the frontier (range: 18.84–48.56) include Algeria, Bosnia and Herzegovina, Ghana, Afghanistan, Morocco, Serbia, Egypt, Bulgaria, Iraq, and North Macedonia. These countries exhibit significantly higher ASMRs at the same SDI level than others. Conversely, the top 10 countries with the lowest ASMRs and hence the minor effective differences at the same SDI level (range: 0.0–0.73) are Singapore, Austria, France, Ethiopia, Somalia, Canada, Peru, Israel, Switzerland, and Puerto Rico (Figure 6B).

### 3.7 Decomposition analysis

**Figure 7:**
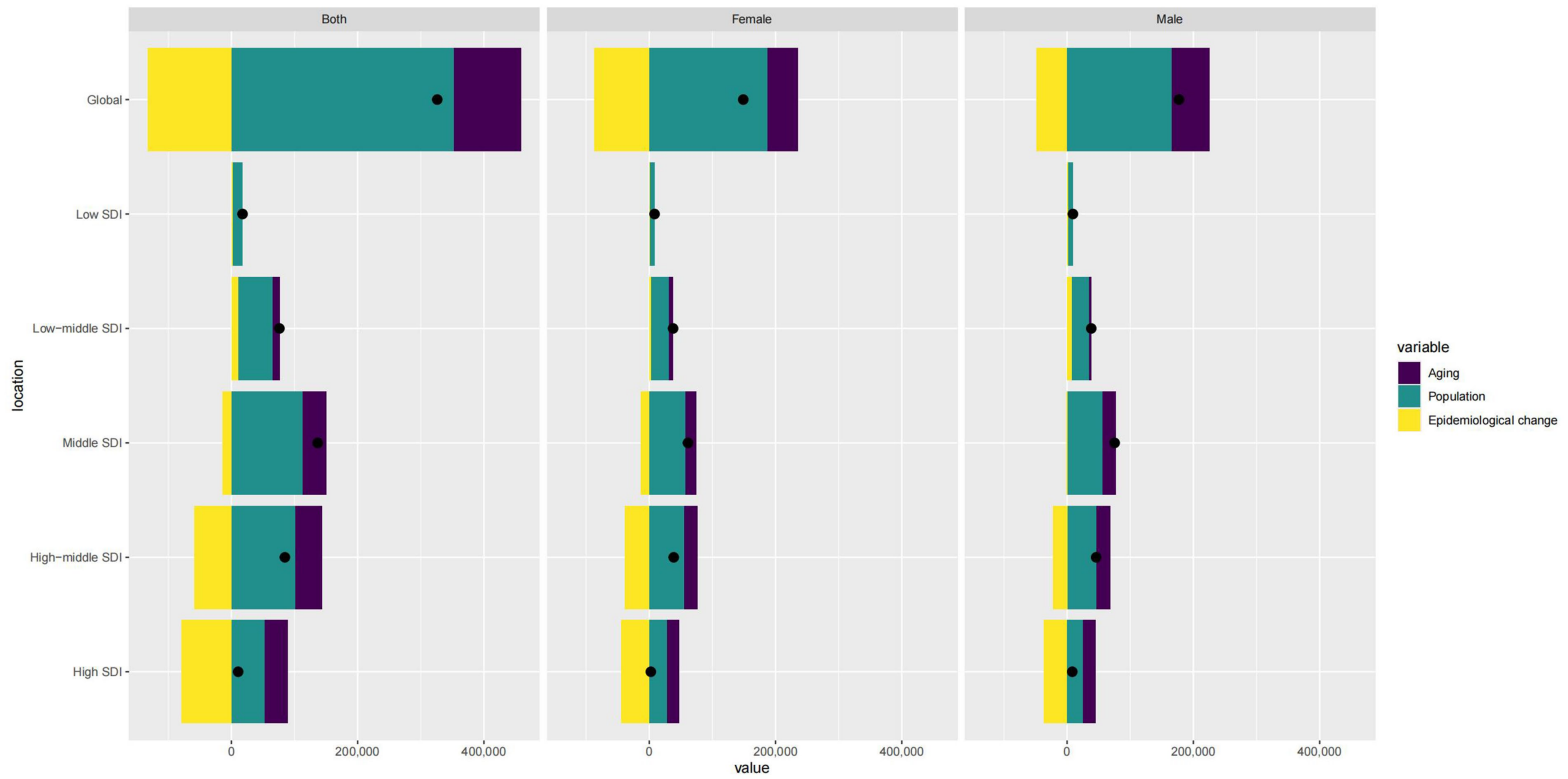
Comparison of contributions from aging, population, and epidemiological changes for different sex groups (Note: Bar charts represent specific contribution values, and points represent relative proportions).

Globally, population growth is the primary contributor to ASMR, followed by epidemiological changes and aging. However, these factors vary significantly by region. Population growth is the dominant factor in areas with Low, Low-Middle, and Middle SDI, with aging gradually increasing in influence. In the High-Middle and High SDI regions, the impact of aging and epidemiological changes surpasses that of population growth (Figure 7).

### 3.8 BAPC forecast

**Figure 8:**
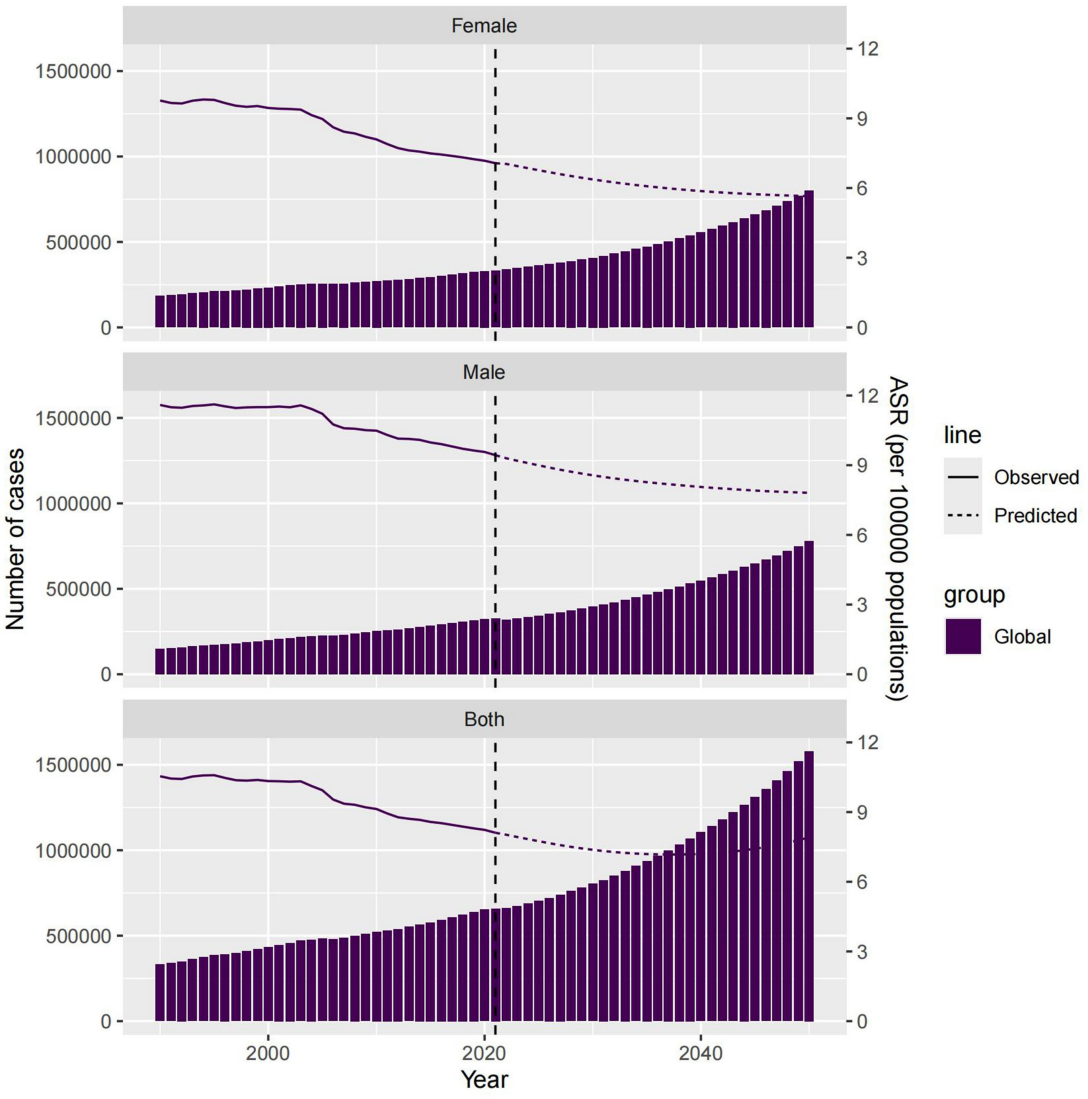
Global and China forecast for deaths and ASMR by sex in 2050.

Finally, we used BAPC to predict the ASMR of IS attributed to HFPG from 2022 to 2050, including a gender subgroup analysis. By 2050, the global absolute number of IS deaths attributed to HFPG is projected to reach 1,579,186.081 (0, 21,632,508.75), with 779,476.9931 (0, 11,257,408.36) in males and 799,709.0882 (0, 10,375,100.39) in females. The ASMR is expected to be 7.910 (0, 178.84), indicating a relative downward trend (Figure 8).

### 3.9 Construction of the shiny platform

**Figure 9:**
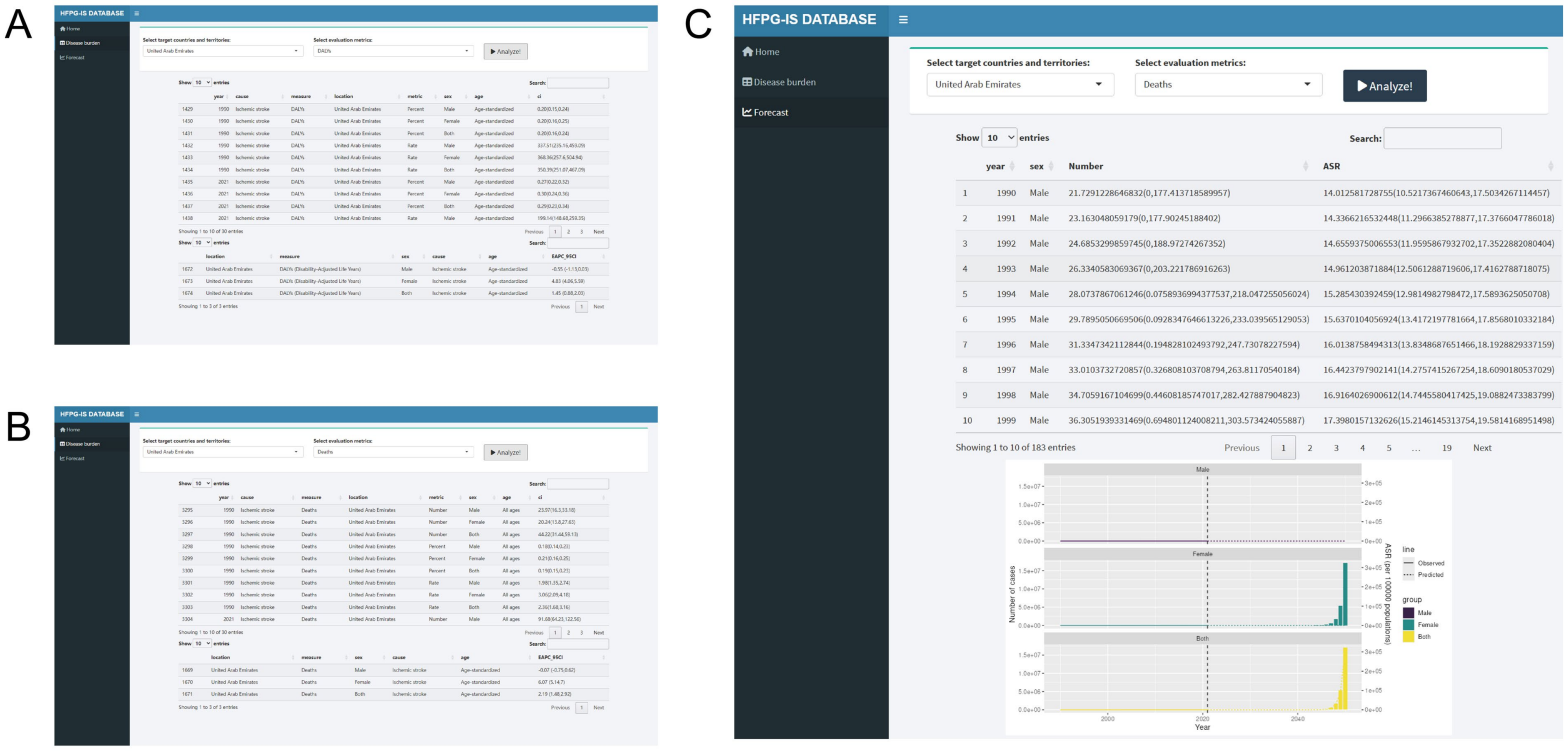
Construction of the HFPG-IHD platform. **(A)** DALYs burden table. **(B)** Deaths burden table. **(C)** Forecasting trends.

We developed the HFPG-IS DATABASE using the Shiny platform to visualize disease burden and forecast results for 204 countries and regions. This platform is freely accessible at http://116.196.73.86:3838/GBD/HFPG-IS/. Previous studies identified that women in the United Arab Emirates exhibit the highest ASDR, ASMR, and corresponding EAPC. To systematically assess the burden and predict future trends in this region, we analyzed the United Arab Emirates using the HFPG-IS platform. EAPC analysis shows a yearly increase in ASDR (overall EAPC: 1.45 [0.88, 2.03]), with the female EAPC significantly exceeding that of males (female: 4.83 [4.06, 5.59], male: −0.55 [−1.13, 0.03]) (Figure 9A). A similar trend is observed for ASMR (Figure 9B). Further, BAPC predictions indicate that by 2050, the number of deaths in the region will rise from 38.52 (0, 233.01) to 17,103,643.90 (0, 29,575,777,215.31), with women contributing the most significantly (Figure 9C).

## 4 Discussion

Globally, the incidence, mortality, and DALYs of IS are on the rise, making it one of the most undertreated severe conditions in the U.S. HFPG plays a critical role, not only increasing the risk of IS but also being closely linked to poor outcomes (19). Therefore, understanding the high-risk populations and their epidemiological characteristics is essential for formulating effective public health strategies aimed at preventing and managing IS attributed to HFPG.

This study is the first to examine the burden of IS attributed to HFPG using global data from GBD 2021. It details the impacts on DALYs and mortality and analyzes variations by region, gender, and age. In different SDI regions, we assessed the effects of age, cohort, and period on ASMR using the APC model and further analyzed the contributions of population, aging, and epidemiological changes. To accurately evaluate the burden across 204 countries and regions, we developed the HGPG-IS platform for visualization.

Initially, we noticed a concerning increase in global DALYs and deaths from IS attributed to HFPG, nearly doubling. However, further rate analysis revealed a contrasting trend, with EAPC for DALYs at −0.715 and for deaths at −0.959, suggesting that the increase is mainly due to population growth, indicating initial control over growth rates. Despite a global easing of the burden of IS attributed to HFPG, regional differences persist. Particularly in Low-middle and Low SDI regions, EAPC results indicate a continued rise in DALYs [Low-middle SDI: 0.51 (0.461, 0.56), Low SDI: 0.241 (0.185, 0.297)] and deaths [Low-middle SDI: 0.482 (0.422, 0.542), Low SDI: 0.287 (0.218, 0.356)], notably in Central Asia, East Asia, North Africa and the Middle East, and South Asia.

To better understand the temporal patterns in disease burden, stratifying the results by decade could provide insights into how medical advancements, such as continuous glucose monitoring (CGM), improved access to insulin therapy, and public health initiatives, have influenced the trends of IS attributed to HFPG. In particular, widespread CGM adoption in high-income countries may have contributed to better glucose control and reduced IS, whereas limited access to essential diabetes care in low-income regions likely exacerbates disparities. A sensitivity analysis accounting for healthcare improvements and a discussion on the potential limitations of temporal trend analysis would strengthen the study’s conclusions.

Differences exist between genders in ASDR and ASMR, with a trend of higher rates in males. Ye et al. (20) examined the global burden of type 2 diabetes from 1990 to 2019. They found that incidence and mortality rates in males have historically been higher than in females, a trend expected to continue. They noted that the 40–59 age group has the highest incidence, though this conclusion is based on all genders. Our study reveals an increasing disease burden among young males, potentially linked to higher rates of overweight and obesity (21). As shown in Figure 3C, even in regions with high SDI, where the disease burden in older populations is declining, it is rising among younger individuals. This suggests a possible adverse impact of metabolic disorders in this demographic. In summary, complications of IS attributed to HFPG, such as stroke, warrant greater attention in young males.

We observed variations in disease burden across regions, particularly in areas with lower SDI, including Low-Middle and Low SDI regions, where the DALYs burden is rising across nearly all age groups, along with mortality. During this period, regions with high SDI have better controlled disease burden. To further explore the correlation between ASMR and SDI, it is evident from Figure 5 that the relationship is not purely linear. Higher SDI often indicates better healthcare conditions, which help control ASMR. However, high SDI regions also experience higher rates of metabolic diseases and population aging, contributing to the burden of IS attributed to HFPG. This was confirmed in subsequent decomposition analysis, showing that higher SDI regions have a more significant proportion of aging populations.

We developed an APC model to elucidate further the roles of various factors such as age, cohort, and period across different SDI regions. Our findings indicate that more recent birth cohorts exhibit better control over ASMR. The high, high-middle, and middle SDI regions have shown the most favorable ASMR trends in the last 5 years. Conversely, in Low-middle and low-SDI regions, the trends are unfavorable, highlighting a significant imbalance in the application of medical advancements, which needs to be better reflected in less-developed areas. Through decomposition analysis, we found that aging and epidemiological changes increasingly outweigh the impact of population growth in the High-middle and High SDI regions. Conversely, in Low, Low-middle, and Middle SDI regions, population growth remains the primary influence, with aging also becoming more significant.

Although the global burden of IS attributed to HFPG is declining and is expected to be well-controlled by 2050, this improvement is mainly seen in high SDI regions. In low SDI regions, the trend is different, particularly among women in the United Arab Emirates. Women there have higher rates of diabetes and metabolic syndrome, with less effective control compared to men (22, 23). Projections indicate a continued rise in the burden among UAE women, warranting attention. Data for other countries and regions can be accessed at http://116.196.73.86:3838/GBD/HFPG-IS/.

In summary, from 1990 to 2021, the global burden of IS attributed to HFPG has increased, with the most significant rise in low and middle SDI regions, including Central Asia, East Asia, North Africa, the Middle East, and South Asia. The 80–84 age group continues to bear the most significant burden for both genders. However, an upward trend in ASMR among younger males (25–49 years) requires additional attention. ASMR is lower in higher SDI areas in low SDI regions, unlike in high SDI regions, possibly due to population aging.

In light of the significant disparities in health outcomes due to IS attributed to HFPG globally, this study advocates for a concerted effort to enhance metabolic adjustments, particularly addressing the metabolic challenges faced by young males. While the worsening of population aging highlights the need to focus on the 80 to 84 age group, it is critical to recognize that age-related stroke vulnerability, such as frailty and comorbidities, should not be conflated with the specific risks posed by HFPG. Therefore, this study emphasizes the importance of early intervention in younger cohorts to mitigate long-term risks. Recommendations include implementing targeted health education programs, promoting healthy dietary habits and lifestyles, and raising awareness about the importance of controlling sugar intake and the risks associated with ischemic stroke. Early health concepts of sugar control should be instilled to prevent IS attributed to HFPG (24). Concurrently, early screening and intervention for blood glucose and other critical indicators are identified as effective strategies for reducing the disease burden (22).

For regions with lower SDI levels, where a larger population faces a more uneven distribution of healthcare resources, there is an urgent need to strengthen medical infrastructure. This is particularly pressing in countries such as Ghana, Algeria, Bosnia and Herzegovina, Afghanistan, Morocco, Serbia, Egypt, Bulgaria, Iraq, and North Macedonia, which bear a disproportionately high disease burden compared to their peers. Governments are urged to increase investment in primary healthcare facilities, including infrastructure development, equipment procurement, and personnel training, to elevate the standard of care. Additionally, fiscal support, talent acquisition, and technological assistance should be leveraged to alleviate healthcare deficiencies and ensure the widespread availability of essential medical services (23).

This study has limitations. The GBD database struggles to apply a uniform standard for accurately assessing disease burden due to varying economic development and healthcare systems across countries. More decisive leadership and political support are needed to monitor and evaluate prevention programs. Additionally, this registry can facilitate the integration of diabetes care across healthcare sectors and strengthen collaboration among researchers, clinicians, policymakers, and patients.

## Data Availability

The raw data supporting the conclusions of this article will be made available by the authors, without undue reservation.

## Footnotes

^1^ https://ghdx.healthdata.org
